# Clinical Features and Predictors of Mortality in Patients With Acute Pulmonary Embolism: A Retrospective Study From Southern Saudi Arabia

**DOI:** 10.7759/cureus.55623

**Published:** 2024-03-06

**Authors:** Usama E Abuelhassan, Ali A Alsalem, Fawwaz A Alshafa, Fahad S Alshahrani, Maram A AlShahrani, Amal K ALAmri, Meaad A Alaqil, Ahmad Ali Al Asim, Eman M Alsultan, Salihah Y Al Mani, Azizah G Badawi, Elham F Alshehri, Eissa A Alshehri, Nour K ALAmri, Abdelrahman M Abdalla, Mervat Khalaf, Tayseer M Ghalyoob, Medhat Elnamaky, Ibrahim M Mahmoud

**Affiliations:** 1 Department of Internal Medicine, Armed Forces Hospital Southern Region (AFHSR), Khamis Mushayt, SAU; 2 Department of Respiratory Medicine, Faculty of Medicine, Cairo University, Cairo, EGY; 3 Department of Internal Medicine, King Khalid University Hospital, Abha, SAU; 4 Department of Medicine, Ministry of Health, Aseer District, Abha, SAU; 5 Department of Radiology, Armed Forces Hospital Southern Region (AFHSR), Khamis Mushayt, SAU; 6 Department of Pulmonology, Beni Seuf University, Beni Seuf, EGY; 7 Department of Critical Care Medicine, Faculty of Medicine, Cairo University, Cairo, EGY; 8 Department of Pulmonary Medicine, Al-Azhar University - Assiut Branch, Assiut, EGY; 9 Department of Critical Care Medicine, Armed Forces Hospital Southern Region (AFHSR), Khamis Mushayt, SAU

**Keywords:** morbidity and mortality, acute pulmonary embolism, disease prediction, clinical features, saudi arabia

## Abstract

Background and methodology: We aimed to investigate the clinical characteristics, outcomes, and mortality predictors in patients with acute pulmonary embolism (PE). Adult patients who were admitted to the Armed Forces Hospital Southern Region, Khamis Mushait, a large tertiary hospital in Southern Saudi Arabia, with the diagnosis of acute PE were retrospectively examined for the predictors of one-year mortality.

Results: The overall in-hospital mortality was 15.6% among 212 patients. In univariate analysis, only age was significantly associated with increased early mortality, whereas age, obesity, presence of active malignancy, hypertension, use of thrombolytics, and Simplified Pulmonary Embolism Severity Index (sPESI) were significantly associated with increased late mortality. By use of binary logistic regression, the presence of obesity (HR 6.010, 95%CI 0.048-16.853, p=0.030), active malignancy (HR 3.040, 95%CI 1.147-8.059, p=0.025), and the use of thrombolytics (HR 8.074, 95%CI 2.719-23.977, p<0.001), were independently significant factors for late (overall) mortality, respectively.

Conclusions: Among Saudi Arabian patients in the Southern Region, our data show that age is an independent factor for increased early and late mortality. The presence of obesity, active malignancy, and the use of thrombolytics, were independently significant factors for increased late (one-year) mortality. These factors should be taken into account for risk stratification and decisions on tailored management of patients with PE. Further prospective multicenter studies are needed.

## Introduction

Pulmonary embolism (PE) is a major cause of hospitalization, morbidity, as well as mortality worldwide [1]. One of the major challenges to the clinician is that most clinical presentations of PE are non-specific, resulting in frequent misdiagnosis [2,3]. Previous studies have demonstrated the utility of different existing prognostic models for acute PE [4,5]. The researchers were interested in developing tools for estimating the risk of medium- and long-term mortality, which is of paramount importance for contribution to clinical decision-making regarding specific/tailored treatment regimes, and optimum follow-up. Despite the availability of data on short-term prognosis, only a few reports investigated the mortality predictors beyond the first 30 days after the insult of PE [6,7].

Characteristically, mortality rates >30% after five years of acute PE have been reported, which corresponds to a 2.5-fold increase in the risk of mortality in comparison to age- and gender-matched general population [8].

A literature review addressed mortality predictors among patients with PE [4-9]. However, there is a considerable lack of such information for patients in Saudi Arabia. Therefore, the current study aimed to investigate the clinical characteristics, outcomes, and mortality predictors in an unselected “real-world” cohort of patients with acute PE, admitted into a tertiary referral hospital, in Southern Saudi Arabia.

## Materials and methods

Study design and participants

This is an observational, retrospective study. The study subjects were all adult (≥ 14 years old) patients admitted to the inpatient units of the Armed Forces Hospital Southern Region (AFHSR), Khamis Mushait, Saudi Arabia, with the diagnosis of acute PE between June 2021 and June 2022. Their data were retrospectively retrieved from the inpatients' electronic records. The enrolment criterion was acute PE diagnosis as per the criteria defined by computed tomography with IV contrast (CTPA). The exclusion criteria were patients who (i) were aged <14 years, (ii) had chronic pulmonary embolism, (iii) had chronic thromboembolic pulmonary hypertension (CTEPH), and (4) had incomplete or deficient follow-up data.

Outcomes and variables

The primary study outcomes of the study were mortality and the associated clinical factors of patients with PE who were diagnosed and treated in our center. The overall mortality was defined as death due to any cause. Early and late mortality were defined as death within 30 days, and >30 days after PE, respectively [10].

The data related to mortality were obtained from hospital records for patients with in-hospital mortality and follow-up (up to one year) using patient ID numbers for those who were discharged from the hospital. A review of the hospital's electronic records was conducted to obtain the baseline features, including those variables utilized to estimate the original and Simplified Pulmonary Embolism Severity Index (sPESI) scores. The following baseline characteristics for all PE cases were recorded: age, gender, body mass index (BMI), obesity (stated as BMI ≥ 30 kg/m^2^), medical and surgical history, laboratory data, initial parenteral or oral anticoagulants used, use of thrombolytics (if any), and the current medications. Other items, like the type of malignancy, other risk factors rather than that of cancer (i.e., history of immobilization or surgery for ≥ 72 hours, and use of hormone replacement or oral contraceptive therapy), concomitant deep venous thrombosis (DVT), type of diagnostic test, treatment for acute PE, history of previous PE, previous DVT, as well as the absence or presence of medical comorbidities were also recorded. PESI and sPESI scores were calculated as previously reported [11,12].

Ethical aspects 

The study was approved by Review Board Committee of the AFHSR (approval number: AFHSRMREC/2022/PULMONOLOGY-INTERANL MEDICINE/565).

Statistical analysis

To examine the compatibility of the numerical variables with normal distribution, the Shapiro-Wilk test was used. The mean and the standard deviation (SD) were used to represent the data fitting the normal distribution, whereas median values were used for those that were not fitting. According to their survival status, the patients were divided into two subcategories: deceased and living. Chi-square analysis was used to examine the association of categorical variables with mortality. The association of normally distributed and non-normally distributed numerical variables with mortality was assessed by the student-T test and the Mann-Whitney U test, respectively. The analysis of the binary logistic regression was used to determine the independent factors determining mortality. P<0.05 was considered statistically significant at a 95% confidence interval (CI). IBM SPSS Statistics for Windows, Version 25.0 (Released 2017; IBM Corp., Armonk, New York, United States) was used for statistical analysis.

## Results

Clinical and demographic characteristics

A total of 212 patients were enrolled in the current analysis, with a median age of 59 years. Of the subjects, 58% were females. Considerable numbers of the enrolled subjects had associated comorbidities. The highest prevalences were reported for hypertension and diabetes mellitus (DM), reported in 45.8% and 41.5% of patients, respectively. According to the PESI score, 46.3% and 53.7% were classified as low-risk and high-risk, respectively, whereas according to s-PESI, 18.9% and 81.1% were classified as low-risk and high-risk classes, respectively. Table 1 details these data.

**Table 1:** Baseline demographic and clinical characteristics of patients with PE (N=212) RR, respiratory rate; PR, pulse rate; SBP, systolic blood pressure; DM, diabetes mellitus; CKD, chronic kidney disease; PESI, pulmonary embolism severity index Data given as n (%), mean ± SD, and median (R), as indicated

Item	Values
Age (years)	
Mean ± SD	58.11 ± 21.3
Median (R)	59 (17-105)
Sex (males), n (%)	88 (41.5%)
Body mass index, mean ± SD	32.7± 7.4
Chronic pulmonary disease, n (%)	7 (3.3%)
Active malignancy, n (%)	12 (5.6%)
Heart failure, n (%)	12 (5.6%)
Prior VTE, n (%)	10 (4.7%)
Unprovoked PE, n (%)	44 (20.8%)
Massive PE, n (%)	2 (1.0%)
Temp. < 36°C, n (%)	39 (18.4%)
RR ≥ 30 minutes, n (%)	6 (2.8%)
PR ≥ 110 BPM, n (%)	76 (36.8%)
SBP < 100 mm Hg, n (%)	34 (16%)
Altered conscious level, n (%)	10 (4.7%)
PaO2 < 90%, n (%)	142 (67%)
Hemoglobin, mean ± SD	12.79 ±2.83
Platelets, mean ± SD	257.2±112.2
Use of thrombolytics, n (%)	6 (2.8%)
Medical comorbidities, n (%)	
Essential hypertension	97 (45.8%)
Coronary artery disease	19 (9%)
DM	88 (41.5%)
CKD	34 (16%)
Obesity	42 (19.8%)
Charlson Comorbidity Index, n (%)	
Mean ± SD	3.23 ± 2.8
Median (Range)	3 (0 - 10)
PESI score, n (%)	
Low risk (classes I-II)	98 (46.3%)
High risk (classes III-V)	114 (53.7%)
Simplified PESI score, n (%)	
Low risk	40 (18.9%)
High risk	172 (81.1%)

Age groups and survival

We categorized the enrolled subjects according to survival among different age groups. Notably, there were significant differences between those with <40 years vs ≥40 years (95% CI 1.333 - 15.548, p=0.010) and those with <60 years vs ≥ 60 years (95% CI 1.108-5.286, p=0.036), respectively. On the other hand, there were no significant differences between those <80 years vs ≥80 years (95%CI 0.989-4.862, p=0.070) (Table 2).

**Table 2:** Different age groups as predictors of overall (one year) mortality

Item	Total, N=212	Survivors, N=179 (84.4%)	Non-survivors, N=33 (15.6 %)	95% CI	P-value
Age group, n (%)				0.989-4.862	0.070
<80 years	163 (76.8%)	142 (87.1%)	21 (12.9%)		
≥80 years	49 (23.2%)	37 (75.5%)	12 (24.5%)		
Age group, n (%)				1.108-5.286	0.036
<60 years	109 (51.4%)	98 (90%)	11 (10%)		
≥60 years	103 (48.6%)	81 (88.7%)	22 (21.3%)		
Age group, n (%)				1.333-15.548	0.010
<40 years	59 (27.8%)	56 (95.0%)	3 (5.0%)		
≥40 years	153 (72.2)	123 (80.4)	30 (19.6)		

Mortality and affecting factors analysis

Mortality follow-up data for one year were completed for all enrolled subjects (n=212). The reported mortality rates were 10.4% and 15.6% for early and late mortality, respectively. The overall in-hospital mortality was 15.6%.

Univariate Analysis

When the factors associated with mortality in patients with PE with regards to the survival and mortality groups were univariate analyzed, only age was significantly associated with increased early mortality (HR 9.227, 95%CI 1.212-70.237, p=0.010), whereas age, obesity, presence of active malignancy, hypertension, use of thrombolytics, and s-PESI, were significantly associated with increased late mortality, respectively.

Characteristically, neither laboratory (hemoglobin level, platelet count, serum levels d-dimer, and troponin I, nor the clot burden quantification upon radiological examination was a predictor of death. Table 3 reveals these results.

**Table 3:** Predictors of early and overall mortality among patients with PE (univariate analysis) VTE, venous thromboembolism; PE, pulmonary embolism; DM, diabetes mellitus; CPD, chronic pulmonary disease; CKD, chronic kidney disease; CAD, coronary artery disease; CTD, connective tissue disease; PESI, pulmonary embolism severity index; s-PESI, simplified PESI

Item	Total (N =212), n (%)	Early (30 days)				Overall (one year)			
		Alive, n (%)	Dead, n (%)	HR (95%CI)	P-value	Alive, n (%)	Dead, n (%)	HR (95%CI)	P-value
Mortality rate		10.4%				15.6%			
Age									
<40 years	59 (28%)	58 (98%)	1 (2%)	9.227 (1.212-70.237)	0.010	56 (95%)	3 (5%)	4.553 (1.333-15.548)	0.010
≥40 years	153 (72%)	132 (86%)	21 (14%)			123 (80%)	30 (20%)		
Gender									
Males	88 (42%)	80 (91%)	8 (9%)	0.786 (0.315-1.962)	0.655	72 (82%)	16 (18%)	1.399 (0.664 – 2.947)	0.443
Females	124 (58%)	110 (96%)	14 (4%)			107 (86%)	17 (14%)		
Prior VTE									
No	202 (95%)	180 (89%)	22 (11%)	0.109 (0.849-0.935)	0.604	171 (85%)	31 (15%)	1.058 (0.772-1.451)	0.657
Yes	10 (5/5)	10 (100%)	0 (0%)			8 (80%)	2 (20%)		
Obesity									
No	170 (80%)	150 (88%)	20 (12%)	0.925 (0.847-1.010)	0.261	140 (82%)	30 (18%)	3.750 (0.933-15.068	0.033
Yes	42 (20%)	40 (95%)	2 (5%)			40 (95%)	2 (5%)		
Active malignancy									
No	200 (94%)	179 (90%)	21 (10%)	0.775(0.818-1.166)	1.000	173 (87%)	27 (13%)	6.407 (1.926-21.317)	0.004
Yes	12 (6%)	11 (92%)	1 (8%)			6 (50%)	6 (50%)		
COVID-19									
No	197 (93%)	175 (89%)	22 (11%)	0.888 (0.845- 0.933)	0.375	165 (84%)	32 (16%)	0.368 (0.047-2.901)	0.476
Yes	15 (7%)	15 (100%)	0 (0%)			14 (93%)	1 (7%)		
Unprovoked PE									
no	168 (79%)	148 (88%)	20 (12%)	0.352 (0.079-1.569)	0.264	139 (83%)	29 (17%)	0.479 (0.159-1.444)	0.244
yes	44 (21%)	42 (95%)	2 (5%)			40 (91%)	4 (9%)		
Massive PE									
No	210 (99%)	188 (89%)	22 (11%)	0.895 (0.855-0.938)	1.000	178 (85%)	32 (15%)	0.305 (0.074 – 1.264)	0.288
Yes	2 (1%)	2 (100%)	0 (0%)			1 (50%)	1 (50%)		
DM									
No	124 (58%)	114 (92%)	10 (8%)	1.8 (0.741-4.374)	0.253	110 (89%)	14 (11%)	2.164 (1.019 – 4.595)	0.054
Yes	88 (42%)	76 (86%)	12 (14%)			69 (78%)	19 (22%)		
Hypertension									
No	115 (54%)	107 (93%)	8 (7%)	1.087 (0.988- 1.197)	0.112	104 (90%)	11 (10%)	2.773 (1.268-6.064)	0.013
Yes	97 (46%)	83 (85%)	14 (15%)			75 (77%)	22 (23%)		
CPD									
No	205 (96%)	183 (89%)	22 (11%)	0.107 (0.851-0.936)	1.000	173 (84%)	32 (16%)	0.100 (0.723-1.340)	1.000
Yes	7 (4%)	7 (100%)	0 (0%)			6 (86%)	1 (14%)		
CAD									
No	193 (91%)	172 (89%)	21 (11%)	0.455 (0.058-3.584)	0.700	162 (84%)	31 (16%)	0.615 (0.795-1.108)	0.744
Yes	19 (9%)	18 (95%)	1 (5%)			17 (93%)	2 (7%)		
CKD									
No	178 (84%)	162 (91%)	16 (9%)	-	0.332	152 (85%)	26 (15%)	-	0.625
Yes	34 (16%)	28 (82%)	6 (18%)			27 (80%)	7 (20%)		
CTD									
No	203 (96%)	181 (89%)	22 (11%)	0.108(0.850-0.935)	0.602	171 (84%)	32 (16%)	0.052 (0.747-1.203)	1.000
Yes	9 (4%)	9 (100%)	0 (0%)			8 (89%)	1 (11%)		
Charlson Index									
None	55 (26%)	53 (96%)	2 (4%)	1.104 (1.021-1.195)	0.071	50 (91%)	5 (9%)	1.106 (0.990-1.236)	0.137
Any	157 (74%)	137 (87%)	20 (13%)			129 (82%)	28 (18%)		
Desaturation									
No	111 (52%)	98 (88%)	13 (12%)	0.737 (0.301 – 1.807)	0.653	95 (85%)	16 (15%)	0.856 (0.457-1.604)	0.706
Yes	101 (48%)	75 (75%)	9 (25%)			84 (84%)	17 (16%)		
Tachycardia									
No	149 (70%)	131 (88%)	18 (12%)	0.493 (0.160-1.522)	0.324	127 (85%)	22 (15%)	0.846 (0.437 – 1.638)	0.679
Yes	63 (30%)	59 (94%)	4 (6%)			52 (82%)	11 (18%)		
Tachypnea									
No	204 (96%)	183 (90%)	21 (10%)	1.245 (0.146 – 10.617)	0.590	172 (84%)	32 (16%)	0.768 (0.091-6.455)	1.000
Yes	8 (4%)	7 (88%)	1 (12%)			7 (88%)	1 (12%)		
Anemia									
No	136 (64%)	126 (93%)	10 (7%)	1.1 (0.987-1.226)	0.063	117 (86%)	19 (14%)	0.758 (0.404-1.425)	0.432
Yes	76 (16%)	64 (84%)	12 (16%)			62 (82%)	14 (18%)		
Thrombolytics									
No	206 (97%)	44 (21%)	162 (79%)	0.984 (0.466-0.955)	0.172	29 (14%)	177 (86%)	12.207 (2.138 – 69.700)	0.006
Yes	6 (3%)	3 (50%)	3 (50%)			4 (67%)	2 (33%)		
PESI									
Low-risk	98 (46%)	92 (94%)	6 (6%)	2.503 (0.939-6.673)	0.072	87 (89%)	11 (11%)	1.891 (0.866-6.129)	0.129
High-risk	114 (54%)	98 (86%)	16 (14%)			92 (81%)	22 (19%)		
s-PESI									
Low-risk	40 (19%)	39 (97%)	1 (3%)	5.424 (0.708 –41.577)	0.085	39 (97%)	1 (3%)	8.914 (1.180 - 67.316)	0.008
High-risk	172 (81%)	151 (88%)	21 (12%)			140 (81%)	32 (19%)		

Binary Regression Analysis

For factors influencing mortality, a multivariate analysis was undertaken. The presence of obesity (HR 6.010, 95%CI 0.048-16.853, p=0.030), active malignancy (HR 3.040, 95%CI 1.147-8.059, p=0.025), and the use of thrombolytics (HR 8.074, 95%CI 2.719-23.977, p<0.001), were independently significant factors for late (overall) mortality, respectively. Table 4 details these results.

**Table 4:** Predictors of late (overall) mortality among patients with pulmonary embolism (binary logistic regression analysis)

Item	HR (95% confidence interval)	P value
Thrombolytic therapy (yes)	8.074 (2.719 – 23.977)	<0.001
Age (≥40 years)	2.277 (0.630 – 8.223)	0.209
Hypertension (yes)	1.457 (0.668 – 3.179)	0.344
Obesity (yes)	6.010 (0.048 – 16.853)	0.030
Active malignancy (yes)	3.040 (1.147 – 8.059)	0.025
S-PESI (high)	4.684 (0.570 – 38.496)	0.151

## Discussion

To the best of our knowledge, only a few studies have addressed the predictors of mortality among patients with acute PE in Saudi Arabia [13]. The current study enrolled 212 patients who were admitted into a tertiary referral center. The obtained results represent the “real-world” demographic characteristics of the population in the Southern Region of Saudi Arabia. Khamis Mushait's elevation is 1998 meters (6555 feet) above sea level, an elevation that is 1478 meters (4849 feet) higher than the average city elevation in Saudi Arabia [14]. This elevation above sea level has its reflections on physiological adaptations and clinicodemographic characteristics of the study populations. 

Considerable numbers of the enrolled subjects had associated comorbidities; 45.8% and 41.5% of patients had hypertension and DM, respectively. These findings highlight the importance of comorbidities and agree with those of Friz et al., who concluded that, unlike the s-PESI score, the Charlson Comorbidity Index (CCI) showed to be an independent prognostic factor for both medium- and long-term mortality [6].

Even though old age is considered one of the risk factors for increased mortality among patients with acute PE [6,15], the results of the current study revealed that patients in younger age groups were at risk for increased mortality, as well. One-fifth of the patients aged ≥40 and ≥60 years died by the one-year follow-up. Again, this could be one of the impacts of a high-altitude environment on the clinicodemographic features of the enrolled patients. The finding of a relatively younger age at presentation in our cohort could be linked to the increase in thrombophilic mutations among Saudi patients as suggested by genotyping of thrombophilic factors [16,17], but further studies in this regard are warranted. 

In the current study, we reported mortality rates of 10.4% and 15.6% for early and late mortality, respectively. The overall in-hospital mortality was 15.6%. Sandal et al. reported higher mortality rates (13.3% at one month, 21.8% at three months, 32.6% at one year, and 51.0% at five years) and justified that by the higher prevalence of malignancy (31.9%) among their cohorts [18].

Mortality analysis of our cohorts revealed interesting results. Univariate analysis revealed that only age was significantly associated with increased early mortality, whereas age, obesity, presence of active malignancy, hypertension, use of thrombolytics, and s-PESI, were significantly associated with increased late mortality. Neither laboratory nor radiological clot burden quantification was a predictor of death. These findings agree with those obtained in a Saudi Arabian study [13]. In their study, Al Otair et al. examined the outcome of PE and the clinicopathological predictors of mortality in patients with PE. They found that tachypnea, congestive heart failure, and tachycardia at presentation were associated with higher mortality. However, radiological quantification of the clot burden was not a predictor of death [13]. These observations, together with our obtained results do not underestimate the importance of radiological findings, but rather they emphasize the importance of clinical factors in predicting mortality among patients with PE.

Despite that s-PESI has been reported previously to have a significant risk prediction among patients with PE [11,12], results of the current study show that other “clinical” and “therapeutic” factors like obesity, active malignancy, and use of thrombolytics are significant predictors of mortality. The multivariate analysis of the current study confirmed that the presence of active malignancy, obesity, and the use of thrombolytics, were independently significant factors for late mortality, respectively.

Clinically apparent VTE was reported to happen in up to 10% of patients with cancer [19]. This could be attributed to the fact that patients with cancer usually have a hypercoagulable state due to the production of substances with procoagulant activity (e.g. cancer procoagulant and tissue factor). The risk of VTE in cancer patients appears to be highest at the time of diagnosis the initial hospitalization, the start of chemotherapy, and at disease progression [20]. 

A review of the literature has found a significantly increased risk for DVT, and/or PE in subjects with obesity, and a reduced risk for underweight subjects, as well as an increased risk of recurrent embolic events once anticoagulation treatment has been stopped/withdrawn [21]. Our observations are in agreement with those of AlEidan, et al., who found that among patients with recurrent VTE, active cancer, and obesity were independent predictors of mortality [22]. Obesity is associated with several factors like raised intra-abdominal pressure, immobility, chronic impairment of fibrinolysis, high levels of fibrinogen, factor VIII levels, von Willebrand factor, and a low-grade inflammatory state [23]. When these factors combine, a prothrombotic state results and elevates the risk of recurrent VTE, and subsequently the mortality rate is increased.

While other studies have shown that some laboratory, radiological, or echocardiographic parameters are predictors of outcome and mortality in patients with acute PE [8-10,13,15], the current study highlights the importance of clinical parameters as predictors of one-year mortality among an unselected “real-world” cohort of consecutive patients with acute PE in Southern Saudi Arabia. Clinicians should be aware of those mortality predictors upon dealing with patients with acute PE. Certain precautions and probably therapeutic management strategies could be undertaken for those patients having these risk factors to avoid unwanted outcomes. 

Despite the limitations inherent to retrospective studies conducted at highly specialized centers, the current study results are robust and provide initial national data that can bridge the existing apparent gap between the daily practice of medicine and academic studies in Saudi Arabia. In patients with the diagnosis of acute PE, it is important to predict those with poor outcomes, and clinicians should be alert to the presence of one or more of those clinical risk factors for increased mortality. Consequently, selecting/tailoring the most appropriate therapy for almost every patient will improve the patient’s outcomes. However, prospective multicenter studies focusing on risk stratification-guided management are recommended for a better level of evidence to strengthen the given practices for the management of acute PE in Saudi Arabia.

## Conclusions

our data show that among Saudi Arabian patients with PE admitted at a large tertiary hospital in the Southern Region, age is an independent factor for increased early and late mortality. The presence of obesity, active malignancy, and the use of thrombolytics were independently significant factors for increased late (one-year) mortality. These factors should be considered for risk stratification and management decisions of patients with PE. Further prospective multicenter studies are needed.
